# Towards the Development of a Device for Assessing the Pliability of Burn Scars

**DOI:** 10.3389/fbioe.2022.856562

**Published:** 2022-06-20

**Authors:** Francesco Dalle Mura, Lapo Governi, Rocco Furferi, Marta Cervo, Luca Puggelli

**Affiliations:** ^1^ Department of Industrial Technology, University of Florence, Florence, Italy; ^2^ Meyer Children’s Hospital, Florence, Italy

**Keywords:** burn scars, 3D measurement, pliability, reverse engineering, additive manufacturing (3D printing)

## Abstract

Burn injuries requires post-accident medical treatment. However, the treatment of burns does not end with first aid because scarred skin must be managed for many years, and in some circumstances, for life. The methods used to evaluate the state of a burn scar based, for instance, on Patient and Observer Scar Assessment Scale or similar ones, often lacks in univocally assessing the scarred skin’s state of health. As a result, the primary aim of this research is to design and build a prototype that can support the doctor during scar assessment, and eventually therapy, by providing objective information on the state of the lesion, particularly the value of skin pliability. The developed tool is based on the depressomassage treatment probe named LPG, currently used to treat burn scars in a number of hospitals. It consists of a non-invasive massage technique using a mechanical device to suction and mobilize scar tissue and is used as a post-operative treatment to speed up the healing process to make the mark of the scar less visible. The prototype is specifically designed to be manufactured using Additive Manufacturing and was validated comparing its performances against the ones of a certified instrument (i.e., the Romer Absolute ARM with RS1 probe). Validation was carried out by designing and developing a tool to put the RS1 probe in the same measurement conditions of the new prototype probe. Tests performed to assess the performance of the devised prototype show that the probe developed in this work is able to provide measurements with a sufficient degree of accuracy (maximum error ±0.1 mm) to be adopted for a reliable estimation of the pliability value in a hospital environment.

## Introduction

The ability of the body to replace damaged cells and repair tissues following an inflammation is crucial, since the target of the healing process is to restore tissue to its original condition. The healing of trauma skin wounds can result in a wide variety of scar types ranging from a fine line to non-aesthetic or pathologic scars. In some cases, tissues completely restore themselves; however, for tissues unable to regenerate themselves, healing is accompanied by the deposition of connective tissue, which typically produces a scar (this term is particularly used to refer to the healing process of a skin lesion) (Vercelli et al., 2003). As widely recognized (Vercelli et al., 2003), a scar is an area of fibrous tissue that replace the normal skin after an injury. It is made up of the same proteins (called collagen) as normal skin, but their arrangement is directional rather than random. Unfortunately, in some cases the scar can be extensive, especially when they are originated by a burn. Therefore, its presence can have a serious psychological impact on the patient, in addition to the physical damage (Zhang et al., 2019). In these cases, first-aid is not sufficient to heal the lesion and long-term post-accident treatments must be applied to pursue the restoration of the normal skin characteristics (Brongo et al., 2012), such as press therapy, intra-lesional steroid injection, radiotherapy, die-laser treatments, silicone device based mechanical massages and surgery (Bignone, 2005). The surgical treatment generally involves the application of autologous dermo-epidermal tissues in the injured area. This complex procedure cannot be applied when the amount of the available skin is limited. This is the case of very extensive scars and of paediatric age patients. For what concerns these last, surgery is even more challenging due to the high risk of disabling and disfiguring scar sequelae (Caviggioli et al., 2008).

Pressotherapy is a physiotherapy method that uses a pneumatic massager to perform sequential compressions in the direction of the circulatory blood flow, assisting venous return to the heart. It has been used to treat many diseases that cause vascular stasis (Güleç, 2009). Steroids are used therapeutically in the management of abnormal scars; however, this is associated with a variety of adverse effects. Their intralesional administration is the most widely used and most effective treatment modality for raised skin scars today (Jalali and Bayat, 2007). The pulsed dye laser and radio therapy is effective in the treatment of a variety of traumatic and surgical scars with improvement in scar texture, colour, and pliability with minimal side effects (Alster and Nanni, 1998). A series of parameters are commonly evaluated to estimate the condition of the scar tissue, which can be related to mechanical properties, physiological properties, aesthetics, and patient perception, to monitor the healing process and the effectiveness of the adopted treatments. Over the years different types of assessment scales have been introduced, all aimed at assigning a numerical score to the injury by rating its characteristics.

For each one of them, the observer (doctor) gives a numerical value within a range (it changes according to the scale) to define the status of the analysed property. When all key characteristics have been rated, it is possible to average the scores and to obtain a burn scar healing index (Giannetti, 2001). Assessment scales differ from each other in terms of analysed/considered parameters and relative range value. Nevertheless, parameters that are considered more relevant are generally recurrent in the principal assessment and are reported in the following: colour, vascularization, thickness or prominence, pliability and elasticity.

The Vancouver Scar Scale (VSS) was the first scale to be defined and is the most recognized and used yet. The physical parameters considered are related to the degree of healing and maturation of the scar, its appearance, but also to the functionality of the skin. Other scales commonly adopted are the Manchester Scar Scale (MSS) and the Patient and Observer Scar Assessment Scale (POSAS) (Beausang et al., 1998; Draaijers et al., 2004). These scales are characterized by limited sensitivity and are only able to detect considerable changes in the state of the same scar. There is currently no absolute gold standard to be used in rehabilitation for the assessment of postsurgical scars, also, to compare scar treatments’ effectiveness or to monitor patient health status, an objective out-come measure should also be responsive enough to capture important changes over time (Vercelli et al., 2003).

An objective assessment methods of skin biomechanical properties could allow to define and compare the effectiveness of treatments more accurately (Vercellia et al., 2015). Objective assessment also facilitates a more rapid definition of the necessary interventions, guaranteeing better results from a functional point of view (Calosso, 2004). For this purpose, in clinical practice, some characteristics of the injured skin are identified, obtaining a set of “universal parameters” for the objective evaluation of health state of a scar as listed in Table 1.

**TABLE 1:** objective assessment parameters for evaluation of health state of a scar.

Parameters	Description
Biomechanical properties	Biomechanical properties of the scar tissue, such as pliability and flexibility, are found to be severely altered compared with the adjacent or contralateral healthy portion
Colour	The colour of a scar depends on melanin (brown pigment produced by activated melanocytes) and erythema (redness due to the presence of haemoglobin within the dilated or re-modelled vessels at the skin level in scars). Colour detection can be complicated by many factors, such as skin thickness, reflection, and other environmental factors such as light and temperature, but also by the patient’s activity and location. Vascularity (assessable by erythema) and pigmentation vary simultaneously, and scar colour is also not entirely uniform
Perfusion	Perfusion, correlated with pigmentation, vascularity, flexibility, and height, is detected by measuring microcirculation. An immature scar has greater blood flow than a mature one. A hypertrophic scar has four times more blood flow than a non-hypertrophic one
Scar size	These properties include area, thickness, and volume
Texture	Scar topology includes appearance or surface roughness
Pathophysiological disorders	Pathophysiological disorders are defined as changes in skin characteristics in terms of transcutaneous oxygen tension, which can be used as an index of maturity in hypertrophic scars and trans epidemic water loss and moisture
Tissue microstructure	The morphology of the tissue presents different characteristics including the microstructure of the tissue, the volume, thickness and density of collagen, the density of the microvasculature, the diameter of the vessels, the spectral characteristics of collagen and elastin fibers and the structure of the capillaries in the scar

When referring to biomechanical properties, one of the most important parameters to be taken into account is the pliability that refers to the elastic texture of the skin and scar. To assess pliability, the observer should touch the skin or scar surface, limiting its use only for clinical assessments; in fact, the difference between normal and damaged skin is considerable, being scar tissue more rigid than healthy skin (Sullivan et al., 1990). Recent studies, scar pliability assessment was performed using the pneumatonometer, in which a small tip contacts and applies suction over the skin surface, and the durometer, which measures the power required to produce deformation of the skin. Both instruments yielded measurements that accurately correlated with Vancouver pliability scores. Therefore, it is possible to establish that pliability is linearly correlated to the scar uplift under suction (Oliveira et al., 2005).

This introduces several issues for patient healing process and, in some cases, can reduce the mobility of the affected area. For these reasons, different therapies are applied to restore the pliability value of the lesion to a normal value (Vercellia et al., 2015) i.e. to reduce the discontinuity surrounding the scar, which equates at best to achieving a value of the scarred skin that is equal to the pliability value of the healthy skin The available procedures for increasing the pliability of the damaged skin can be roughly classified as “no-suction” and “suction” methods (Moortgat et al., 2016; Choo et al., 2021).

One example of no-suction methods is ElastiMeter (Rodrigues and Fluhr, 2020), a device produced by Delfin, which measures skin pliability by using extensometers without altering skin structure based on skin deformation induced by forces of the instrument’s probe: an indenter is mounted on a flexible support to which extensometers are connected. When a pressure is applied, support deformation is recorded. Other methods implements high-frequency ultrasound and/or ultrasound electrographic methods to quantify the stiffness of hypertrophic burn scars. Unfortunately, up to now, only a few studies have investigated whether EM can be used to evaluate the clinical activity of pathological scars, and their conclusions are inconsistent (Hang et al., 2021).

“Suction” methods, instead, measure skin displacement under the application of negative pressure, by perform several application/rest cycles (Rodrigues and Fluhr, 2020). Among the “suction” devices developed until now, the most interesting solutions are Cutometer (Busche et al., 2018), DermaLab probe (Gankande et al., 2015; Elrod et al., 2019) and Nimble (Elrod et al., 2019). All of them have found more use in cosmetics than clinical procedure (Sulaiman et al., 2016).

On the other hand, for several years now, in clinical practice tissue mechanostimulation techniques have been used in rehabilitation medicine and in reconstructive and aesthetic plastic surgery (Majani and Majani, 2013). The most used mechano-stimulation technique, called Depressomassage or LPG [named after its inventor Louis Paul Guitay (Fodor, 1997)], is a non-invasive massage technique using a mechanical device to suction and mobilize scar tissue. It is used as a post-operative treatment to speed up the healing process to make the mark of the scar less visible. This therapy stimulates the regeneration of connective tissue through a mechanized massage (Vercellia et al., 2015). This operation is carried out using dedicated equipment, which puts the treatment area under depression and, through the action of a pair of rollers (motorized for adult patients, idle for pediatric), exerts a rolling and unrolling movement of the skin, which is known as “skin gymnastics”. This movement stimulates cells to produce collagen and elastin (Moseley et al., 2007; Waibel et al., 2013).

Whichever approach is adopted to strengthen the pliability of the patient’s skin, an objective assessment has only been studied to a limited extent in the scientific literature. As a result, new insights into methods and systems for qualitative, objective, and reliable measurement of such a critical parameter are sought.

Accordingly, the present paper proposes a tool able to support the clinician in performing an objective and real-time measurement of the skin pliability. The proposed device, in particular, is capable of measuring the value of skin displacement during LPG massage and consists of an appropriately designed hardware comprising a modified LPG system with a distance sensor embedded within. Such a system, from now on named “LPG-D”, is meant to be used as an alternative to the current LPG device. Of course, the pliability assessment is not only dependent on the usage of the proposed device; other factors, such as the employment of other no-contact methods or ultrasonic echo-equipped ways, should be considered. As a result, it is critical to emphasize that the primary goal of this research is to provide an additional objective instrument useful for investigating burn scar pliability.

## Materials and Methods

As mentioned above, the idea proposed by the authors, in collaboration with Meyer Children Hospital, is to devise a system able to perform a reliable measurement of the damaged skin pliability. More in detail, this system integrates the traditional LPG probe with an appositely devised real-time measurement device. The main advantage in embedding the measurement system with the depressomassage device is that during the LPG therapy the doctor will be able to measure the effect of suction directly during the treatment. Accordingly, the pliability is measured in real time, and any improvement reached during a session can be recorded and compared to the previous sessions. Moreover, using the proposed system the therapy procedure commonly adopted by the doctors does not change and no additional time is required.

The first step of the study consists of the choice of the most appropriate sensor to acquire the skin raising during the LPG treatment. The LPG-based Device (LPG-D) is designed once the most reliable sensor is selected. The subsequent phase consists of the development of a measurement device to be embedded on a commercial high precision 3D scanner. The sole purpose of the definition of such a prototype (named “comparison prototype”) is to assess the LPG-based device performance by means of a comparison between the data acquired with the two different systems. In fact, as explained below, the “comparison prototype” allows acquiring more precise data on the patient skin, thus allowing to retrieve the ground truth for the assessment of the LPG-based device.

### LPG-Based Device: Sensor Selection

**FIGURE 1:**
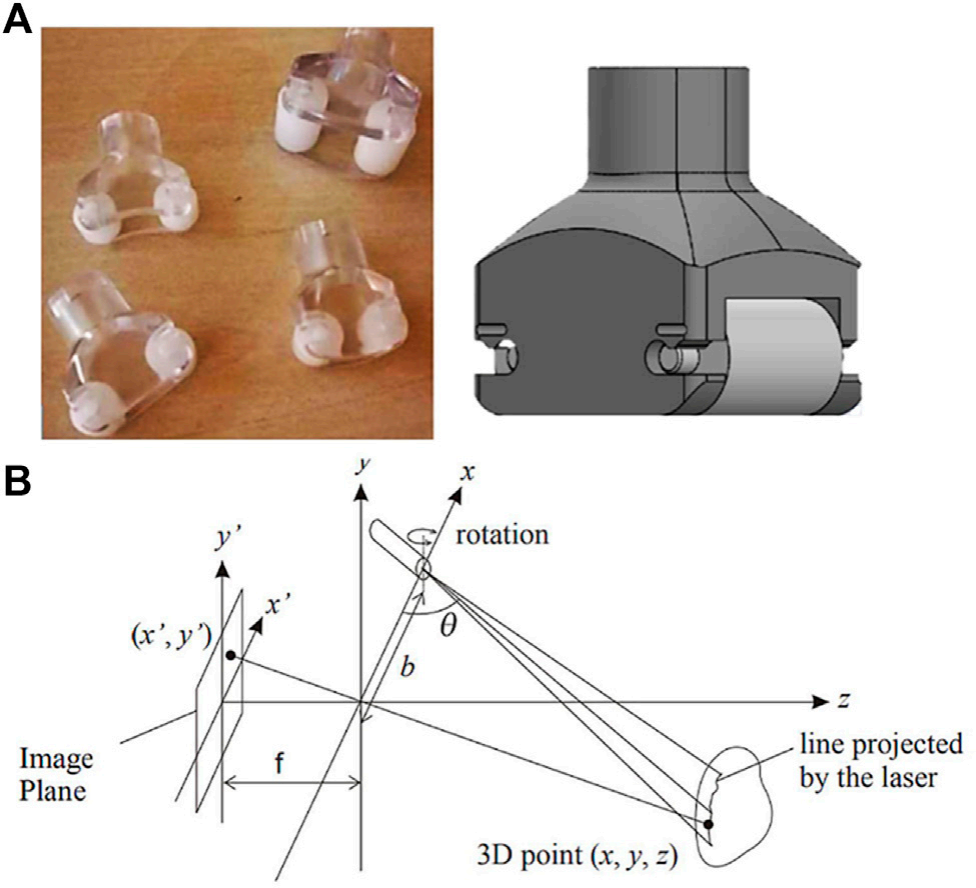
**(A)** LPG device used at Meyer Children Hospital; **(B)** geometry of triangulation technique: the lens are located at the origin, with focal length ƒ to the image plane, and baseline b between the projector and the camera.

The concept of the prototypal device takes inspiration from of the traditional LPG device, currently used at Meyer Children Hospital, in terms of overall design solution (see Figure 1). It consists of a device equipped with two idle rollers and a connection for an air suction tube in a vertical position. The head used for massage is manufactured in different sizes (large, medium, small) depending on the patient’s body size. In our case study, the device with smallest dimensions was considered since it consists of a device commonly used for the treatment of both paediatric and adult patients. Such a device has a rectangular base of 23 mm × 30 mm and a height of 33 mm (Figure 1A).

In order to measure the pliability, the prototypal device is required to include a proximity sensor to measure skin raising under the applied suction force. To this aim, a first set of specifications is defined based on the dimensions of the selected LPG probe. In particular, the following parameters are defined:

• Measurement Range: from 20 to 40 mm.
• Resolution: < 0.1 mm.
• Dimension: < 50 mm × 50 mm × 50 mm.

Therefore, an extensive market research was carried out with the aim of finding a solution that match the requirements. Fortunately, three potential solutions were identified, all of which are based on the laser-camera triangulation technique (see Table 2).

**TABLE 2:** List of sensors technical characteristics (*repeatability).

Code	Producer	Operation	Range (mm)	Resolution (µm)	Dimension (mm)		
					Width	Height	Depth
Q4XTKLAF100-Q8	Banner	Triangulation	25–100	200	33.5	57.5	18
OM70-L0070.HH065.VI	Baumer	Triangulation	30–70	1	26	74	55
ILD1420-25CL1	Micro-Epsilon	Triangulation	25–50	1*	46	30	20
ILD1420-50CL1	Micro-Epsilon	Triangulation	35–85	2*	50	60	20.4
OD2-N30W04U0	Sick	Triangulation	26–34	2*	44.4	31	17
OD1-B035H15U25	Sick	Triangulation	20–50	6*	44.4	31	17
OD1-B035H15U14	Sick	Triangulation	20–50	6*	44.4	31	17
OD1-B035H15I25	Sick	Triangulation	20–50	6*	44.4	31	17
OD1-B035H15I14	Sick	Triangulation	20–50	6*	44.4	31	17
OD1-B035C15I25	Sick	Triangulation	20–50	6*	44.4	31	17
OD1-B035C15I15	Sick	Triangulation	20–50	6*	44.4	31	17
OD1-B035H15A15	Sick	Triangulation	20–50	6*	44.4	31	17
OD1-B150F0AQ14	Sick	Triangulation	50–250	200*	44.4	31	17
CP08MHT80	Wenglor	Angular-Measurament	30–80	8	50	41	20
IL-030	Keyence	Triangulation	20–45	1*	37.9	48.5	22.6
VDM18-100/32/105/122	PEPPERL + FUCHS	Triangulation	30–100	0.1% range	50	50	17
OMT100-R100-2 EP-IO-V31	PEPPERL + FUCHS	Triangulation	40–100	0.1	44.5	21.5	11
FT 10-RLA-60-PNSL-KM4	SensoPart	Triangulation	10–70	<100*	21.1	14.6	8

**FIGURE 2:**
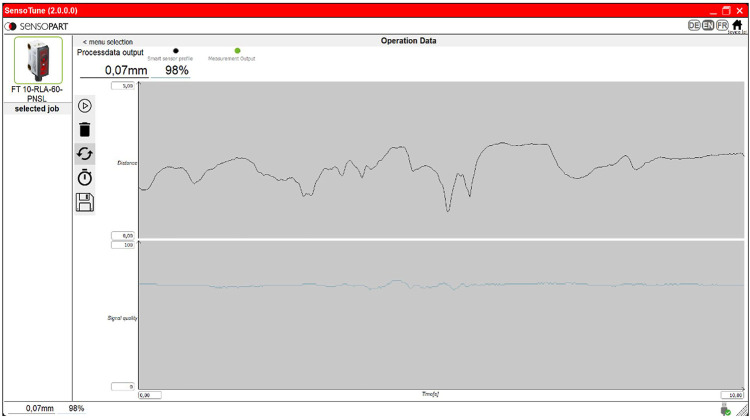
Screen of the sensopart program: the numerical value of the measured distance and the quality of the signal are shown on the top left. Graphs of these parameters as a function of time are shown in the centre.

Based on the technical specifications listed in Table 2, the product FT-10 RLA-60-PNSL-KM4, produced by SensoPart Industriesensorik GmbH, proves to be the best option being its dimensions significantly smaller than the ones of competitors with similar performances. It consists of a distance sensor based on laser-camera triangulation. A diode (transmitter) projects a laser point on the target. If its surface is not specular, the light is reflected in a diffused way, so that part of the reflected lights is captured by optical receiver (optical sensor and lens array). The position of the laser point (defined in the sensor coordinate system) is retrieved applying the triangulation principle (Moortgat et al., 2016). Finally, the distance between laser point and the reference one (generally coincident with the origin of the sensor coordinate system) is calculated. An example of the triangulation system configuration is in Figure 1B. Together with the FT-10 RLA sensor, two software are provided to interface the sensor with a PC. The first one allows users to regulate all the sensor’s parameters (e.g., process data output and signal quality level..., etc.), showing in real time the numerical distance value on screen. The second one is dedicated to the (partial) management of the measurement settings. Moreover, the distance value is graphically represented as a function of time. To optimize the measurement process, it is possible to use both software simultaneously, obtaining a complete management of the parameters and the graphic visualization of the output results. This data acquisition process would let the doctor observe time-series measurements in the graphical interface, making the skin pliability estimation a simple and immediate process (see Figure 2).

### LPG-Based Device Design

**FIGURE 3:**
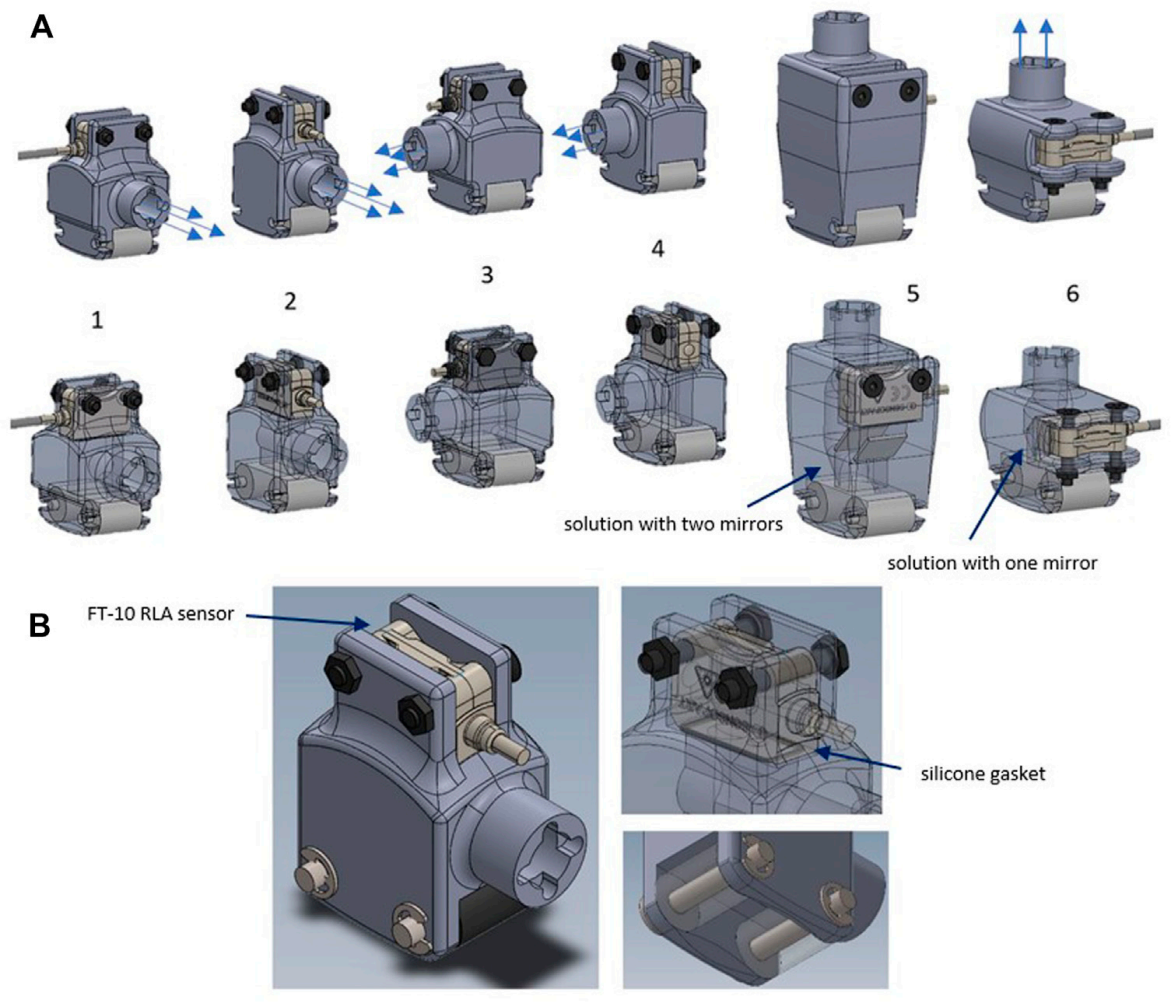
**(A)** Different design solution for prototypal device; **(B)** selected solution of prototype probe for LPG massage.

As mentioned above, the LGP head used in paediatric patients’ treatment has two idler rollers and a connection for an air suction tube. In addition to these two features, the devised prototype is required to securely host the sensor in an appropriate position for assuring the correct acquisition of the skin raising during treatment. Based on the dimensions of the distance sensor, six design solutions were initially devised, changing reciprocal orientation of sensor, rollers, and air suction tube (see Figure 3A).

Constructive solutions from 1 to 5 differ from each other due to the reciprocal orientation between sensor, air intake and rollers. In solutions number 5 and 6 the air intake connection is vertically placed. To allow the sensor’s laser beam to reach the optimal acquisition point one (in solution number 6) or two (in solution number 5) mirrors are placed inside the probe body.

Among the designed solutions, the second one was considered optimal because the rollers, air intake and sensor are all directed in the same direction. As a result, the connection cables are easier to manage, and the movement during therapy is smoother.

The suction connector is placed on the side and the sensor is placed on the upper part maintained in position by two screws with nuts. A silicone gasket is inserted to prevent leakage in the contact area between the sensor and the body. Figure 3B shows the constructive solution described before.

The body of the prototypal device was manufactured by means of 3D printing technology. The printer used for the realization of the prototypal device is the FormLab Form 3 model. This printer uses LFS (Low Force Stereolithography) printing technology. The main reason for selecting this technology is the component’s sealing. In fact, with other 3D printing technologies processing polymers, such as Fused Deposition Modeling (FDM), perfect sealing cannot be guaranteed without performing post-printing treatments on the piece. In fact, leakages are a consequence of the presence of empty space between the passage of material deposition and the adjacent one, or between two consecutive layers (FormLab Form 3)^1^.

### Comparison Prototype Design

Despite FT-10 RLA sensor is designed to meet industrial requirements, no certification is provided about its performances in terms of accuracy and reliability. This means that data acquired using the devised system have to be validated against a ground truth to prove their effectiveness. Therefore, a “Comparison Prototype” (CP) based on certified laser camera triangulation probe was designed. To this purpose, Romer Absolute Arm 7520 3D scanner with RS1 laser triangulation camera probe is chosen as the device, which allows sufficiently accurate 3D measurement that can be considered a ground truth for the specific application. This scanner provides a three-dimensional map of the scanned area, thus allowing its use for different scar characteristics evaluation, including skin raising.

The 3D scanner also includes the following features:

• Both arm and probe are certificated, according to ASME B89.4.22 and VDI/VDE 2617-9^2^
• Accuracy is 2 sigma/30 µm and the minimum point spacing (mid-range) is 0.014 mm.

It is important to highlight that the CP is far more precise with respect to the LPG-D since it uses a high-resolution Laser-camera triangulation system. Unfortunately, it cannot be used during the therapy since both the 3D acquisition and the post-processing of acquired data cannot be performed in real-time. For this reason, the development of this prototype has the sole purpose of assessing the performance of the LPG-based system.

**FIGURE 4:**
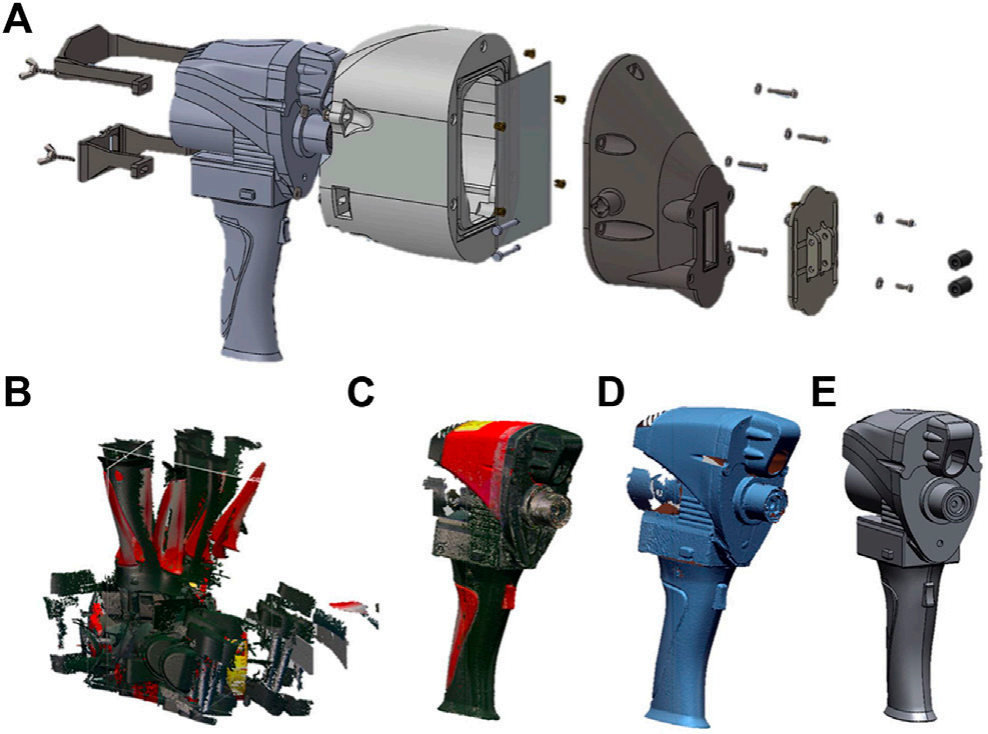
**(A)**test device: (from left to right) blocks for axis fixing, RS1 probe, head rear part, screen glass, head front part, rollers seat, idle rollers; **(B)** NextEngine scans imported to Geomagic^®^ Design X™; **(C)** combine the various scans of the RS1 probe through the “volume merge” function; **(D)** texture elimination; **(E)** CAD geometry modelling of the probe.

The CP is designed with the same skin interface of the LPG-based measurement system in terms of dimensions. Furthermore, as already stated, it is required to be attached on the Romer Absolute Arm 7520 3D scanner with RS1 laser triangulation camera probe. The inside chamber has a “watertight” compartment to create vacuum and restrict air leakage and it is necessary that the point of intersection between the laser beam and the camera (focus zone) is positioned on the skin surface between the inner rollers (see Figure 4A).

The design procedure followed for the realization of the test device provides the 3D scanning and reconstruction of the RS1 probe using reverse engineering methods. The first step is the creation of a 3D relief of the RS1 probe. The 3D geometry of the RS1 probe was acquired using the NextEngine Desktop 2020i 3D scanner, based on MultiStripe Laser Triangulation (MSL) camera technology.

Every time the NextEngine scanner acquires an image, it generates a file containing a point cloud, or the mesh of the object, which can be imported into the Geomagic® Design X™ program to perform the reconstruction. In the Geomagic® Design X™ program the meshes obtained from the single scans are editable and distinguishable from each other, so that it is possible to eliminate, for each of them, the parts generated by noise or by the presence of contour elements on the object. The scans were eventually aligned and blended to create a single piece reflecting the mesh of the RS1 probe (see Figures 4B–E).

**FIGURE 5:**
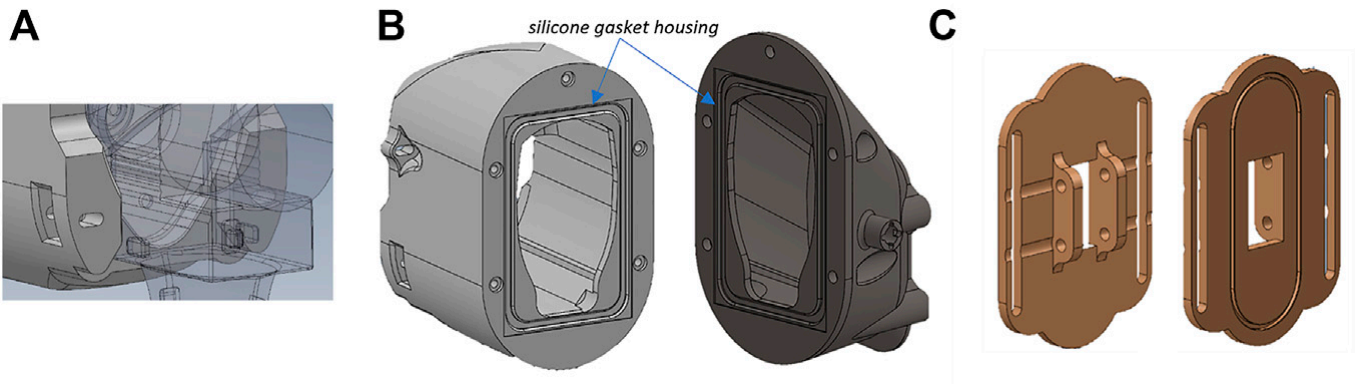
**(A)** Component “Rear Head Part” coupling with the probe RS1; **(B)** “Rear Head Part” and “Head Front Part”; **(C)** Roller Seat component.

Through these operations, it was possible to obtain a CAD model of the RS1 probe as shown in Figure 4D. Then, an error analysis between CAD and polygonal mesh was conducted to identify the reconstructed areas with deviation over a tolerance value. Based on the CAD geometry, obtained by the aforementioned procedure, the “Rear Head Part” was designed. The internal cavity of the component was obtained through an offset of the external surfaces of the RS1 probe, in this way a perfect fit is guaranteed as shown in Figure 5A.

The “Head Front Part” (see Figure 5B) connects the test device to the air intake system through a coupling. The geometry of this component considers the viewing angle of the Romer’s RS1 camera and the direction of the laser beam, ending near the optimal point of acquisition. A glass screen is placed between these two elements to reduce the volume of air suctioned by the pump. Silicone gasket seats are used to prevent air leakage from one chamber to the other.

**FIGURE 6:**
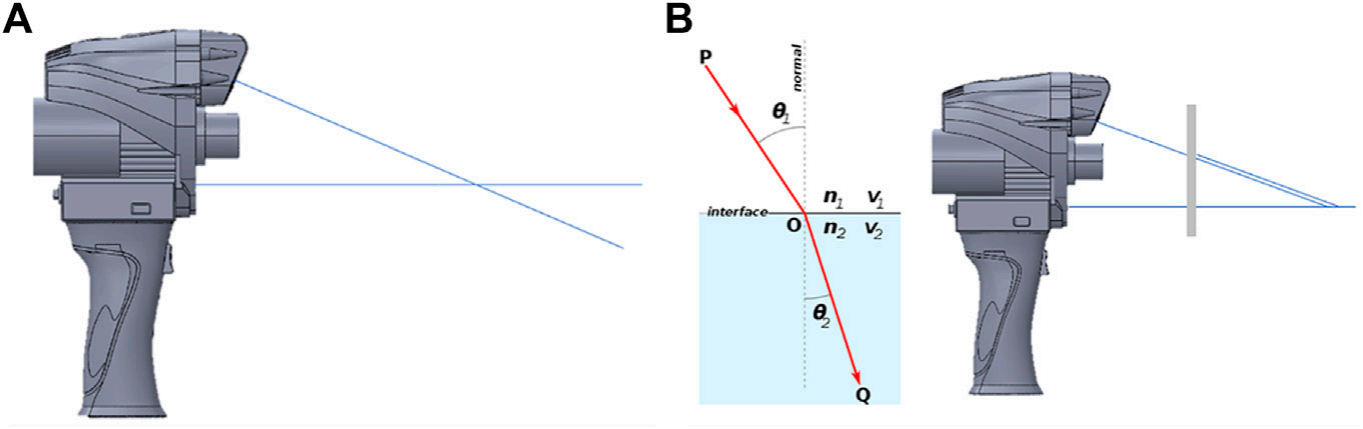
**(A)** Intersection point between laser beam and camera optical axis; **(B)** Snell’s Low and effect on the RS1 probe with screen glass.

Another silicone gasket is mounted at the interface between the “Head Front Part” and the “Roller Seat” component (see Figure 5C). This last element has two side eyelets and the roller housing (same dimensions of the hospital device); with this constructive solution, it is possible to modify the positioning of this element to match the acquisition area centre with the laser beam and chamber intersection point. Two elements called “Axial fixing Block” are located in the probe’s rear area and are connected to the component “Rear Head Part” to prevent disassembly. Due to the presence of a glass screen the different refractive index of the material compared to air induce an error in 3D optical triangulation. To investigate this effect, the distance of intersection between the laser beam and the camera optical axis was estimated using the geometry of the prototype and the probe (screen glass thickness, positioning, and angle of incidence) and the physical parameters of the glass (refractive index), as shown in Figure 6A.

Snell’s law states that the ratio of the sines of the angles of incidence and refraction is equivalent to the ratio of phase velocities in the two media, or equivalent to the reciprocal of the ratio of the indices of refraction (see Figure 6B).

$$\frac{sin\theta_{2}}{sin\theta_{1}}=\;\frac{v_{2}}{v_{1}}=\;\frac{n_{1}}{n_{2}}$$

The orthogonality between the glass screen and the camera axis simplifies the system due to the absence of refraction effects. In this case, it is sufficient to apply Snell’s law only to the axis of the laser beam, obtaining a deviation in the axis perpendicular to the glass screen of 0.64 mm.

To confirm the hypothesis, experimental testing was carried out. To this task, a specimen with known dimensions was measured with and without glass screen. Once scanned the object with the Romer Absolute Arm RS1 scanner, reference planes were extracted from the planar surfaces of the model and from the base plate. The process was repeated by introducing the glass screen and for a different configuration of the object. Subsequently, the differences in terms of normal distances between the same reference planes obtained with the scans with and without the glass screen were calculated.

The test was executed for different configurations obtaining as a result an independent deviation value from the acquisition distance and the glass screen position. It is possible to assert that the presence of the glass screen does not generate any considerable disturbance during the measurement of the skin pliability value.

After these considerations, the RS1 probe adaptor was realized with 3D printing technologies. Some components were realized with Formlab Form3 printer while others with a FDM printer. Moreover, silicone gaskets have been inserted between the different components to avoid air leaks in areas of no interest, and the whole adapter has been painted black to avoid the inside laser reflection effect.

### Validation Procedure of the LPG-Based Device

As previously mentioned, a validation test needs to be performed to verify the accuracy and repeatability of the LPG-D system. Test consists in acquiring the same specimen using both the LPG-D and the CP devices. Acquired data are then compared, taking into account that the 3D data acquired by the CP are the ground truth for the measurement. In particular, the validation is assessed in two different conditions: without suction, for determining the sensitivity of the proposed device, and with suction, for mimicking the actual working conditions.

**FIGURE 7:**
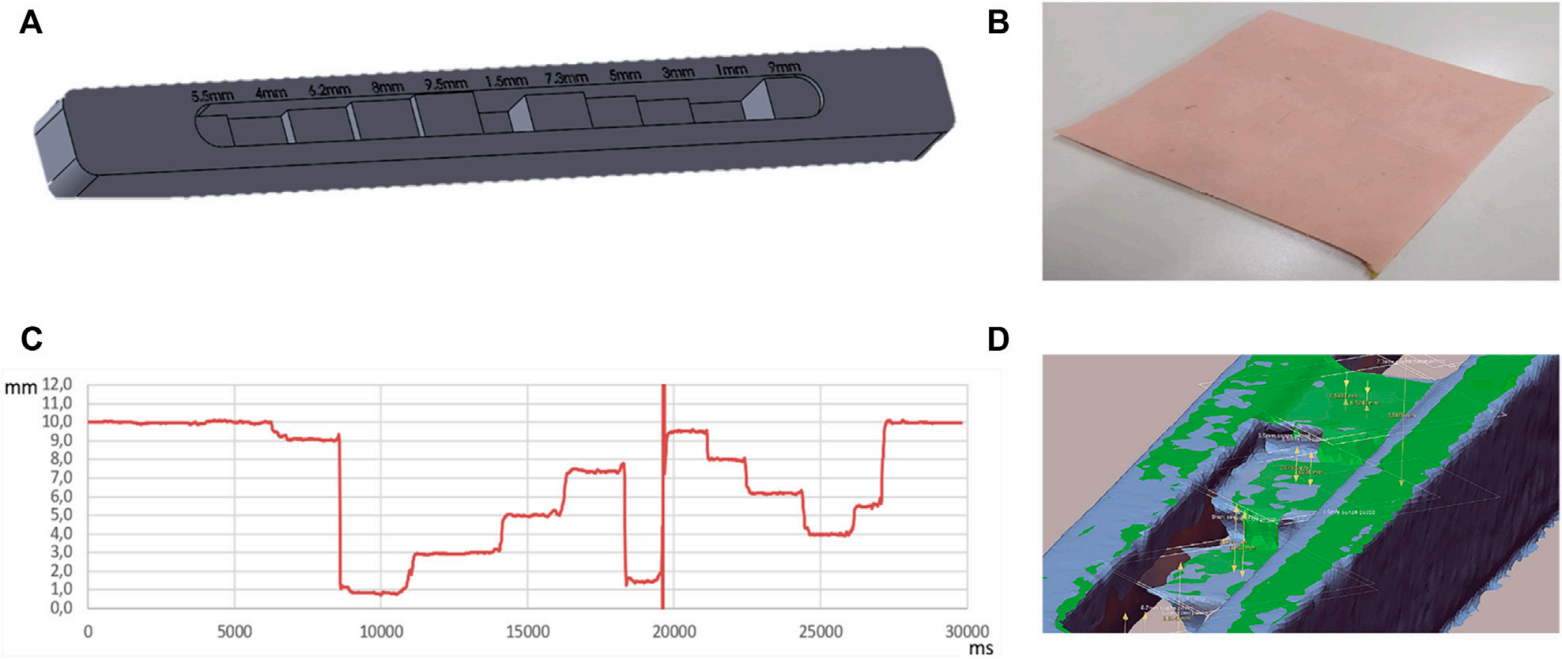
**(A)** Ad hoc specimen for LPG-D sensitivity test; **(B)** Silicon skin specimen; **(C)** Ad-hoc step height evaluation with LPG-D; on the x-axis the time value in milliseconds and on the y-axis the distance in millimetres; **(D)** Plan generation and distance evaluation. Comparison between scans with and without the adapter (in green with the adapter in blue without).

Two distinct specimens are created to validate the measurement system. The first one, depicted in Figure 7A, is developed for no-suction tests. It was realized in photopolymer resin (Black FLGPBK04) which has the following properties: Flexural modulus 2.2 GPa, Modulus of elasticity 2.8 GPa, Elongation 6.2%. (FormLab Form 3). It presents grooves with variable depth spanning from 1 to 9 mm. The resin block accuracy with different steps is equivalent to the layer thickness (equal to 25 microns). The comparison between the measurement of the depth of these grooves using, respectively, the LPG-D and the CP systems is carried out and the results are presented in the next Section.

In particular, four acquisitions of the specimen were performed for the sensitivity evaluation of the LPG-D without suction. The acquisition was carried out by sliding the device on the ad hoc test specimen from one side to the other with a speed approximately of 0.01 m/s. The measurement test was performed by connecting the FT-10 sensor to the PC and, through the dedicated program, were acquired the numerical values of the depth of the steps as a function of time. From the measurements carried out on the specimen, it is possible to derive the following statistical parameters:

• Mean value: the mean value for each interval calculated without taking in consideration the presence of noise peaks.
• Median: calculated on the interval of points.
• % median error over value: percentage error that indicates the deviation between the value obtained with the median and the theoretical value.
• Mean value on 50 points: the mean value has been evaluated only on 50 points centered on the selected interval; in this way it is possible to avoid including in the calculation the transient zones that present high noise peaks.
• Average threshold ± 0.2 mm: the average value per interval was calculated considering the points that did not deviate by ± 0.2 mm from the previously assessed median value.

The second specimen consists of a simple silicone sheet with 1 mm thickness (see Figure 7B). In fact, such a specimen is able to reproduce, according to Meyer Hospital surgeons, the mechanical behaviour of the skin (Uchida et al., 2016). Performance comparison between the LPG-D and the PC devices are conducted during suction; to reproduce the same conditions as during LPG treatment a comparable hospital pump is used in this work. In addition, the pump used for the study has a manometer to measure the depression generated inside the chamber. For both cases, tests were performed three times to obtain enough data to evaluate the repeatability of the measurement. Two different tests were carried out to assess the performance of the devised LPG-D using suction: Steady state time evaluation and measurement repeatability evaluation.

**FIGURE 8:**
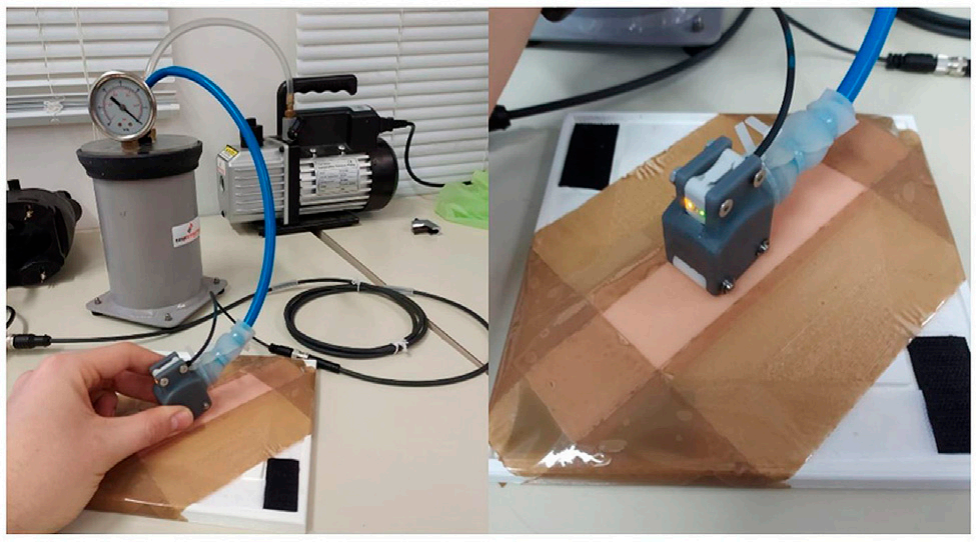
Test bench for steady state time evaluation and repeatability evaluation tests.

The first test was performed by placing the prototypal device on the silicon skin specimen activating the air suction system and moving the sensor towards a line with a speed of, approximately, 0.01 m/s (see Figure 8).

The second test was performed by placing the sensor on a section of the specimen and cyclically activating the vacuum pump. Results obtained during the aforementioned tests are described in the next Section.

## Results

This section presents the results obtained by acquiring the two aforementioned specimens using the two different devices; this allows drafting some consideration on the reliability of the newly conceived LPG-D.

### Sensitivity Test on the LPG-Based Device Without Suction

Referring to the results obtained for the sensitivity evaluation of the LPG-D without suction, Figure 7C shows the first (out of four) measurement results acquired by the distance sensor embedded in the system. Moreover, in the first and last part of the graph in Figure 7D the measured distance value is around 10 mm; this is caused by the fact that the sensor is not positioned in correspondence of a step, but it is in the initial or final part of the specimen. The vertical asymptote located around 20,000 ms is due to the sudden transition between two steps with different heights. In the real application of the sensor, this event is very uncommon, because the skin and scar surface does not present discontinuities of this type. Consequently, this phenomenon is not a critical factor of the measurement system. As it is possible to observe from the graph, all the zones present micro-oscillations around the value of the step height; therefore, to evaluate sensitivity and precision, it is necessary to calculate some statistical parameters. Table 3 lists the results for the above parameters obtained for the four repetitions.

**TABLE 3:** Data of four tests evaluated for the LPG-D sensor sensitivity test.

	Step height (mm)	9	1	3	5	7.3	1.5	9.5	8	6.2	4	5.5
1	Mean value (mm)	9.1	0.89	2.98	5.06	7.25	1.49	9.55	7.95	6.23	4.02	5.55
	Median (mm)	9.08	0.85	2.95	5.01	7.37	1.45	9.5	8	6.18	3.97	5.49
	Error % median over value	0.219	4.494	1.006	0.988	1.655	2.684	0.523	0.628	0.802	1.243	1.081
	Mean value on 50 points (mm)	9.04	0.83	2.95	4.98	7.36	1.46	9.48	8	6.17	3.97	5.48
	Average threshold ± 0.2 mm (mm)	9.06	0.90	2.96	4.99	7.3	1.47	9.49	7.99	6.18	3.97	5.5
2	Mean value (mm)	9.1	0.84	2.96	5	7.36	1.42	9.5	7.96	6.18	3.93	5.5
	Median (mm)	9	0.83	2.92	4.93	7.265	1.38	9.42	7.94	6.1	3.89	5.45
	Error % median over value	1.098	1.190	1.351	1.4	1.290	2.816	0.842	0.251	1.294	1.017	0.909
	Mean value on 50 points (mm)	9.01	0.91	2.98	4.98	7.34	1.48	9.47	8.01	6.15	3.96	5.47
	Average threshold ± 0.2 mm (mm)	9.04	0.94	2.97	4.97	7.33	1.47	9.47	7.98	6.17	3.95	5.5
3	Mean value (mm)	9.09	0.88	3.04	5.08	7.37	1.5	9.6	8.04	6.13	4.05	5.58
	Median (mm)	9.08	0.87	3.02	5.03	7.37	1.48	9.55	8.08	6.16	4.02	5.58
	Error % median over value	0.110	1.136	0.657	0.984	0	1.333	0.520	0.497	0.489	0.740	0
	Mean value on 50 points (mm)	9.03	0.85	2.94	5.01	7.35	1.5	9.53	8.02	6.17	3.97	5.55
	Average threshold ± 0.2 mm (mm)	9	0.87	2.94	4.96	7.28	1.49	9.48	7.98	6.15	4	5.51
4	Mean value (mm)	9.05	0.89	3.07	5.11	7.37	1.51	9.54	8.2	6.26	4.1	5.48
	Median (mm)	9.09	0.88	3.03	5.05	7.41	1.5	9.56	8.09	6.29	4.035	5.49
	Error % median over value	0.441	1.123	1.302	1.174	0.542	0.662	0.209	1.341	0.479	1.585	0.182
	Mean value on 50 points (mm)	8.99	0.84	2.96	5.01	7.36	1.46	9.46	8.02	6.19	3.99	5.48
	Average threshold ± 0.2 mm (mm)	9.02	0.9	2.99	5	7.31	1.48	9.51	7.98	6.18	3.98	5.49

From these data, it is possible to calculate average values for each statistical data (see Table 4) obtaining reference values for test device comparison as shown in the following table. In order to have a consistent amount of data for the LPG-D sensitivity test, scans of the specimen were performed by changing the scanning direction and acquisition speed. The scanning direction was changed by switching the specimen positioning: the measurement was realized starting from the 5.5 mm high step or the 9 mm high step. Regarding the acquisition speed, it is directly controlled by the operator who manually scans with the LPG-D. To achieve different speeds, the operator slides over the specimen slower or faster maintaining a constant pattern as possible. The sensor parameters were unchanged for all tests.

**TABLE 4:** Average statistical parameters evaluated for the LPG-D sensitivity test; ad-hoc step height evaluation using the RS1 scanner with and without adapter.

Average statistical parameters evaluated for the LPG-D sensitivity test											
Step height (mm)	9	1	3	5	7.3	1.5	9.5	8	6.2	4	5.5
Mean value (mm)	9.085	0.875	3.012	5.062	7.337	1.48	9.547	8.037	6.2	4.025	5.527
Median (mm)	9.062	0.857	2.98	5.005	7.353	1.452	9.507	8.027	6.182	3.978	5.502
Error % median over value	0.467	1.986	1.079	1.136	0.87	1.874	0.524	0.679	0.766	1.146	0.543
Mean value on 50 points (mm)	9.017	0.857	2.957	4.995	7.352	1.475	9.485	8.012	6.17	3.972	5.495
Average threshold ±0.2 mm (mm)	9.03	0.9	2.965	4.98	7.305	1.477	9.487	7.982	6.17	3.975	5.5
Ad-hoc step height evaluation using the RS1 scanner with and without adapter											
Grooves depth (mm)	1	9	7	5	2.7	8.5	0.5	2	3.8	6	4.5
CP measured distance from top best-fit plane and step best-fit plane (without adapter and with adapter) (mm)	0.99	9.11	7	5	2.7	8.6	0.56	2.06	3.88	6.06	4.58
	1	—	—	5	2.7	—	0.52	2.02	3.85	—	4.57
Difference (mm) between CP and actual values	0	—	—	0	0.1	—	0.04	0.06	0.03	—	0.01
FT-10 (mm)	9.085	0.875	3.012	5.062	7.337	1.48	9.547	8.037	6.2	4.025	5.527
RS1 scanner (mm)	9.01	0.89	3	4.99	7.28	1.4	9.44	7.94	6.12	3.94	5.42
Difference (mm)	0.075	0.015	0.012	0.072	0.057	0.08	0.107	0.097	0.08	0.085	0.107

Since the median value automatically eliminates measurement peaks caused by noise, it represents the parameter that most closely represents the actual value of the grooves height (Romer Absolute Arm Top Features)^3^.

The groove heights were compared with the results obtained using the CP device, which is able to reconstruct the entire specimen geometry as a polygonal mesh. To obtain an average distance value, the best-fit plane of the scanned step geometry was created and the distance to the top best-fit plane of the specimen was measured. A scan of the specimen was made both with and without the screen glass to provide additional verification of the negligibility of the refractive effect.

Table 4 shows the mean values of the distances between the best-fit planes obtained by four different measurements and compared with the actual dimensions of the specimen. It can be observed that the difference between the scans with and without glass is negligible. Some data are absent in the table; this is due to the fact that the Romer Absolute Arm 7520 RS1 scanner, which is a laser-camera triangulation scanner, is not able to acquire information on too high cavities. Moreover, the test device must be perpendicular to the specimen during the scanning phase affecting the evaluation of deep steps. Nonetheless, the CP proves to be accurate enough to be considered the gold standard for this type of measurement and can thus be used as ground truth for the LPG-D. Table 5 compares the measurements performed with the CP and the LPG-D devices, respectively. Comparison shows that the LPG-D error in measuring the grooves height is lower than 1 mm. Such a value is acceptable for the clinical practice.

**TABLE 5:** Comparison between mean values obtained by means of FT-10 sensor and Romer Absolute Arm 7520 RS1 scanner.

FT-10 (mm)	9.085	0.875	3.012	5.062	7.337	1.48	9.547	8.037	6.2	4.025	5.527
RS1 scanner (mm)	9.01	0.89	3	4.99	7.28	1.4	9.44	7.94	6.12	3.94	5.42
Difference (mm)	0.075	0.015	0.012	0.072	0.057	0.08	0.107	0.097	0.08	0.085	0.107

### Test on the LPG-Based Device With Suction

Observing the results of the measurement carried out on the silicon skin specimen of Figure 8, a time interval of about 3 s can be estimated to establish a constant level of depression. Moreover, after 5 s the synthetic skin reaches a plateau corresponding to roughly 2.25 ± 0.05 mm.

**FIGURE 9:**
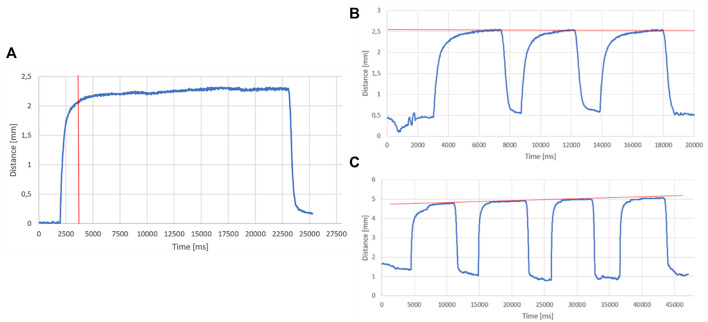
**(A)** Distance value trend measured by the LPG-D as a function of time; **(B)** distance trend on silicone specimen; **(C)** distance trend on healthy skin.

Therefore, it could be concluded that to obtain an uninfluenced evaluation of the suction effect transition it is necessary to wait for at least more than 3 s from the initial lifting effect. Even if the device slides along the skin the suction regime condition persists (see Figure 9A).

For what concerns the results obtained in the second test on synthetic skin, it is possible to notice that the distance exponentially increases immediately when the suction is activated and then stabilizes near a distance value of 2.5 mm and a pressure of 0.1 bar (see Figures 9B,C). The same test was also performed on a healthy forearm skin section obtaining an average deviation of 4.9 mm with a depression of 0.12 bar. The red line indicates the trend of the maximum deviation value for each repetition within the same cyclical test. The results obtained by operating under suction conditions show a gradual decrease of the baseline height after each cycle. This effect can be attributed to the fact that the prototype is maintained in position through manual placement, which does not guarantee a constant pressure of the device on the skin. The fact that the zero is variable may simply be related to the applied pressure of the operator who generates sensitive micro-movements for the prototype maintaining a nearly fixed position.

In the real application of the sensor, this phenomenon is completely unlikely, because the pump is not activated intermittently: this does not represent a real criticality of the measurement system. Moreover, the maximum cycles have an increasing trend; this can be justified by the fact that the section of skin subjected to the test remains the same for the entire test duration. More specifically, the section of skin suffers intermittent traction actions caused by suction. The tissue increases its pliability generating the effect like the LPG massage.

In addition, the CP system was tested during suction. The suction effect estimation of such a device was realized by connecting the pump to the “Head Front Part” component. The test was performed on the silicone specimen by scanning it once without activating the pump, then with the pump activated. The section of silicone skin was acquired by moving the adapter, connected to the scanner, from one direction to another maintaining the rollers connected to the skin.

**FIGURE 10:**
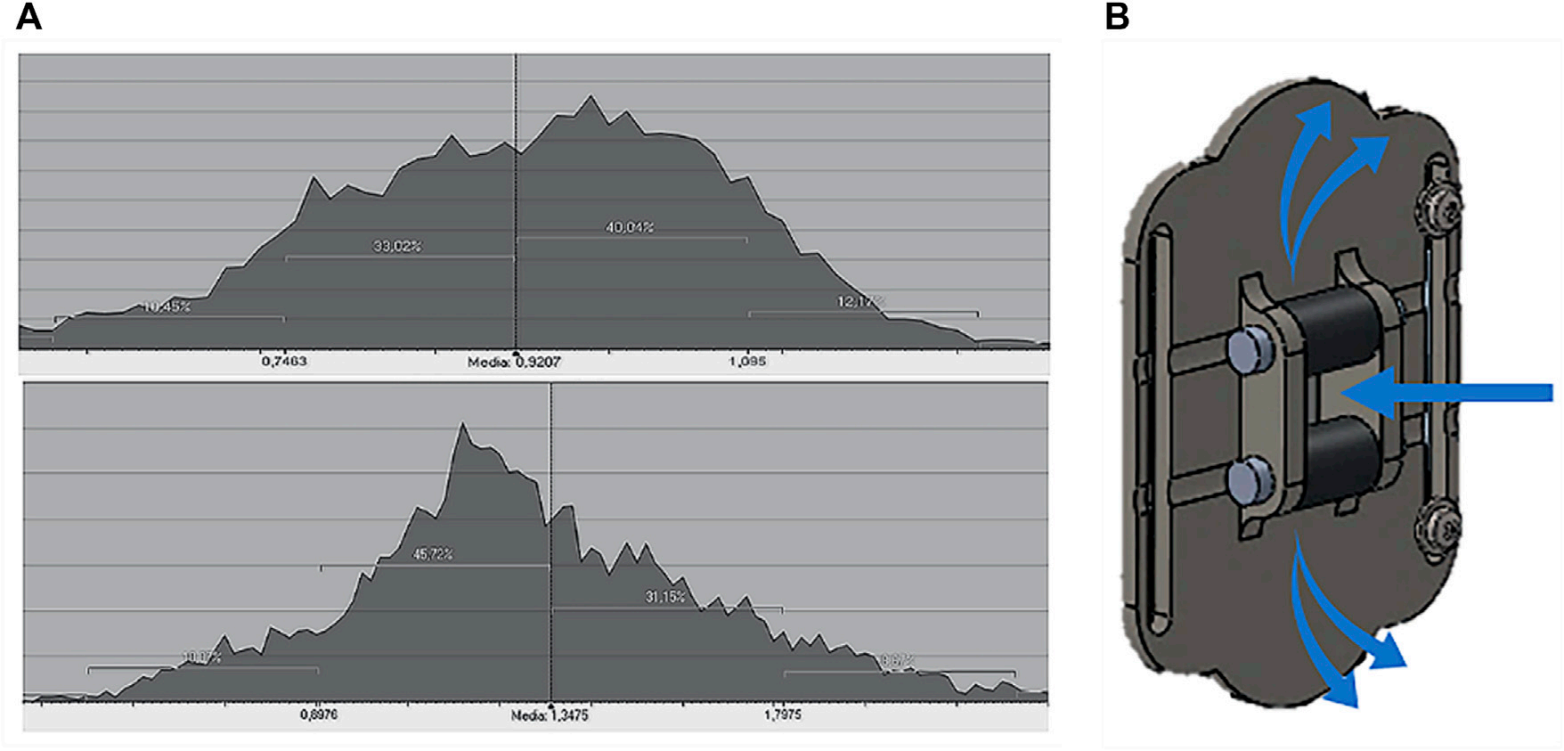
**(A)** Mesh deviation histogram: test on silicone skin and healthy skin; **(B)** leakage effect between idle rollers and “roller seat” component.

An analysis to evaluate the punctual distance between the two scans was carried out using Geomagic® Design X™. In addition to a graphical visualization, such a software package provides the deviation histogram between the two polygonal meshes. The average value of the distance could be extracted from this graph, which resulted 0.92 mm on silicon skin test and 1.34 mm for healthy skin test (with a 0.05 bar depression, see Figure 10A).

This test shows that the test device has a non-negligible leakage effect. This problem comes from the fact that the idle rollers used on this prototype are made with 3D printing and do not present a perfect adhesion with the surrounding components (see Figure 10B). This has impact on the overall raising of the synthetic skin which is lower than the one obtainable with a lower (or null) leakage. However, it is worth noting that the suction test is performed with the same depression value as the traditional LPG massage. This pressure can be adjusted for instance to take into account the leakage effect and based on the thickness of the skin being treated. In fact, because of the loss of skin adnexa, some sections of the burned area may be difficult to suction. As a result, greater effort is required to plicate the tissue when there are more skin adhesions (hence, in the case of the machine more suction is needed, and the choice of a smaller cannula size is recommended). In any case, also in presence of leakage, the sensor may is able to appropriately measure the skin displacement, even if in this case the pliability is lower than expected.

## Conclusion

The implementation of an objective rating system for burn scar pliability would bring significant change to current clinical practices. This is particularly true when dealing with pliability assessment. The current methods for measuring such a parameter are based on the visual assessment of the skin raising, i.e., by using subjective approaches that does not allow for an accurate and repeatable evaluation, as well as on other non-contact methods or on ultrasonic echo-equipped devices. Since the LPG treatment proved to be effective in increasing the skin pliability of burned area of patients (Worret and Jessberger, 2004), the ambition of this work was to propose a system able to perform such a therapy and, at the same time, to measure the amount of skin that is raised by the depression caused by the LPG-D. In this way, the clinician can be aware of this important parameter for the assessment of the pliability of the patient burned area in real-time. Accordingly, in this paper a prototypal LPG probe to be used for performing the treatment and for measuring the pliability in real-time. In fact, the new device, named LPG-D, combines the LPG probe, currently used at Meyer Children Hospital, and a real-time pliability measurement system.

The process began with the creation, through 3D printing technologies, of the hospital device with an integrated distance measuring system. Due to the different common use of this sensor, there was no certification for the required use; for this reason, different tests were carried out for the evaluation of its sensitivity and for assessing the repeatability of the measurement. Moreover, a comparison device called comparison prototype was developed to simulate the same measurement conditions as the LPG-based one, but with a better distance measurement sensor (Romer Absolut Arm RS1 scanner).

Test results demonstrated that despite the designed LPG-D has a lower accuracy with respect to the CP, its measurements can be considered sufficiently accurate for the specific application. In detail the system is able to accurately measure the skin displacement, as demonstrated by tests on the specimen With this solution, during LPG therapy, the doctor will be able to measure the effect of suction, estimating the pliability in real time, and he will be able to report any improvements compared to the previous sessions. Moreover, the therapy procedure adopted by the doctors will not be modified and no additional time will be required.

Because of pandemic emergency from Covid-19, it was not possible to perform any kind of tests on Meyer Children Hospital patients, or on volunteers, with burn injuries, therefore, the tests were performed on healthy skin sections or on silicone skin. In order to further validate the prototype created, it is necessary to perform the tests described above on patients with burn scars and evaluate the results of therapy over time. The clinical trial could then eventually bring about the implementation of this prototype into clinical practice without altering LPG therapy procedure.

## Data Availability

The raw data supporting the conclusions of this article will be made available by the authors, without undue reservation.

## Footnotes

1
https://formlabs.com/it/materials/engineering/

2
https://www.hexagonmi.com/it-IT/products/portable-measuring-arms/absolute-arm-7-axis

3
https://www.hexagonmi.com/it-IT/products/portable-measuring-arms/absolute-arm-7-axis
